# Physical Health Challenges, Healthcare Needs, and Barriers to Care among Firearm Injury Survivors: A Mixed Methods Analysis

**DOI:** 10.1007/s11524-025-01049-9

**Published:** 2026-01-26

**Authors:** Daniel C. Semenza, Nazsa S. Baker, Devon Ziminski, Jeanna M. Mastrocinque, Darnell Whye, Lesly Heredia

**Affiliations:** 1 Department of Sociology, Anthropology, and Criminal Justice, Rutgers University, Camden, NJ USA; 2 Department of Urban-Global Public Health, School of Public Health, Rutgers University, Piscataway, NJ USA; 3 New Jersey Gun Violence Research Center, Rutgers University, Piscataway, NJ USA; 4 The Wraparound Project, School of Medicine, University of California, San Francisco, CA USA; 5 Department of Justice Studies, Rowan University, Glassboro, NJ USA; 6 Center for Family Services, Cure4Camden, Camden, NJ USA

**Keywords:** Firearm injury, Gun violence, Healthcare access, Functional disability, Substance use

## Abstract

Firearm injury survivors often face significant physical, mental, and logistical challenges following their injury, yet research on their healthcare needs remains limited. This mixed methods study examines health challenges, healthcare needs, and barriers to care among 107 firearm injury survivors in a small Northeastern city. The project emerged from a collaboration between university researchers and community-based violence prevention specialists. Participants completed a cross-sectional survey assessing physical health, functional disability, healthcare access, and demographic factors. A subsample of 15 survivors also participated in semi-structured interviews. Quantitative results show that a majority of survivors experience pain, require regular medical treatment, and report functional impairments that interfere with daily life. Respondents report high levels of unmet healthcare needs, including access to affordable care, specialists, and primary providers. Barriers to care include cost, lack of insurance, unreliable transportation, and difficulty navigating the healthcare system. Qualitative interviews illustrated the toll of post-injury pain, trauma, and anxiety, while also highlighting resilience, self-directed care, and changes in care-seeking behavior. Survivors voiced concerns about medication access, potential addiction, and logistical burdens. The findings underscore the complexity of survivor needs and point to opportunities for hospital-based violence intervention programs and trauma recovery centers to play a larger role in post-injury care coordination. This study highlights the value of participatory, community-engaged research in illuminating the lived experiences of firearm injury survivors and advancing care models that address both structural and personal barriers to recovery.

**Supplementary Information:**

The online version contains supplementary material available at 10.1007/s11524-025-01049-9.

## Introduction

In 2023, nearly 47,000 people died from firearm-related injuries [1]. Estimates suggest that non-fatal shootings occur two to four times more frequently than fatal shootings in the USA, although exact figures remain unclear due to the absence of comprehensive national data from official sources [2, 3]. Firearm injury survivors face an array of health challenges including subsequent psychiatric disorders, substance use disorders, pain diagnoses, and shifts in health behaviors (e.g., sleeping, eating, exercise) [4–7]. Survivors also confront difficulties accessing healthcare systems, barriers to follow-up care, and financial impediments, particularly related to lack of insurance or underinsurance [8]. Those who have been recently shot often experience traumatic stress and anxiety about returning to communities where violence remains a persistent threat after they are discharged from medical care [9–11]. Shooting survivors face the most immediate and severe risks to their well-being within a much broader ecosystem of those indirectly affected by firearm violence nationwide [12].

Despite a growing body of research on the healthcare needs of firearm injury survivors, there remain opportunities to expand this work. Most existing studies rely on rather small samples, likely due to the significant challenges of working with firearm injury survivors, who may be unable to respond to research requests or fearful of retribution from their attackers. Prior studies have often relied upon the use of descriptive clinical data regarding injuries and symptoms, rather than highlighting patient needs through direct surveys and interviews post-discharge in the community [13–15]. No studies to our knowledge have leveraged a mixed methods approach that combines quantitative survey data from a large sample of shooting survivors with in-depth interview data from a smaller sub-sample of survivors. With important exceptions [16, 17], few studies regarding the health needs of firearm injury survivors have been conducted on the basis of collaborative partnerships between university researchers and community violence prevention specialists (CVPS) in local communities. These partnerships provide critical expertise necessary for working with survivors of firearm violence and documenting their unique needs, emphasizing the experience of those most intimately familiar with the challenges faced by survivors [18].

To address these gaps in prior research, the current study offers a mixed methods analysis of the health challenges, healthcare needs, and barriers to care among a quantitative sample of 107 firearm injury survivors and a sub-sample of 15 interviewees. The study resulted from a collaboration between university researchers and a local team of community-based violence prevention specialists to shed light onto the significant challenges faced by a unique population that is frequently overburdened yet underserved.

## Methods

### Study Design

We used a convergent mixed-method approach in this study, including a cross-sectional survey and qualitative interviews with adults injured by a firearm in a small Northeastern city. All participants (*N* = 107) completed a survey about their health, access to healthcare, and experiences of violence alongside demographic information. Figure A1 in the Appendix outlines the mixed methods convergent design. A subset of participants (*N* = 15) then participated in a qualitative interview that focused on the circumstances of their injury and their healthcare experiences post-injury. This study used a community-informed, participatory approach, emphasizing an inductive investigation of our research questions rather than a priori expectations [19]. Community partner researchers were involved in each step of the research process including the development of the research questions, participant recruitment and data collection, data analysis, and assistance with writing the manuscript. The mixed methods design allowed us to analyze the complementary data and corroborate findings across quantitative and qualitative methods [20].

### Sample and Recruitment

Our university-community partnership was instrumental to the success of this study. Recruitment was conducted in partnership with the staff at the local community violence intervention (CVI) organization, who recruited participants through purposive sampling throughout their day-to-day work. Participants were included regardless of how long ago they had been shot. Many individuals did not want to discuss the details of their injury so this information was not used to screen participants. We note that CVPS staff members involved in recruitment for this study were compensated through their organization for this additional labor and belonged to the same local communities from which participants were recruited. Potential participants were asked about participation while community partners were in the field, e.g., in the hospital, on the street, and at the organization’s headquarters. Surveys were collected using Qualtrics on an electronic tablet or via paper-and-pencil, depending on participant preference. Community partners assisted in the development of a recruitment strategy and were available to help participants read and interpret the survey if needed.

The study’s purpose and procedures were explained to each potential participant, and informed consent was obtained from each person. Participants received a $25 gift card. The survey took approximately 20–30 min to complete. Survey respondents were then asked if they wanted to complete an interview for an additional $25. Each interview was semi-structured and lasted approximately 30 min. Interviews were conducted by two members of the team (one university and one community researcher) and audio was recorded. Survey data from the paper surveys was entered into an online form using Qualtrics and cleaned and coded for analysis. Interview audio data was transcribed verbatim and then coded for analysis. All study procedures were reviewed and approved by the Rutgers University Institutional Review Board. University-affiliated researchers were CITI certified while the community researchers were trained through the Community Involvement in Research Training program [21].

### Materials and Measures

The survey included questions about personal health, experiences with the healthcare system, violence, childhood experiences, and demographic information. We collected demographic information from all participants including the following: gender identity, race, ethnicity, employment status, education, income, relationship status, and age.

We examined various *health challenges* among shooting survivors. Measures were derived from the World Health Organization’s (WHO) Quality of Life – Brief Scale (WHOQOL-BREF) [22]. The scale has been validated as a reliable means of assessing general quality of life in diverse settings and we focus here on three health-related items [23–25]. First, we asked participants, “How satisfied are you with your health?” with responses ranging from “very dissatisfied” to “very satisfied.” Second, we asked, “To what extent do you feel that physical pain prevents you from doing what you need to do?” with responses ranging from “not at all” to “an extreme amount.” Third, respondents were asked, “How much do you need medical treatment to function in your daily life?” with responses ranging from “not at all” to “an extreme amount.”

We assessed for functional disabilities using six items derived from the American Communities Survey and the US Census Bureau [26–28]. Participants were asked whether they were currently experiencing any of the following six functional disabilities: (1) hearing difficulty: deafness or having serious difficulty hearing; (2) vision difficulty: blindness or having serious difficulty seeing, even when wearing glasses, (3) cognitive difficulty: having difficulty remembering, concentrating, or making decisions; (4) ambulatory difficulty: having serious difficulty walking or climbing stairs; (5) self-care difficulty: having difficulty bathing or dressing; and (6) independent living difficulty: having difficulty doing errands alone such as visiting a doctor’s office or shopping.

To assess *healthcare needs* in the survey, participants were asked, “What are the resources or services you think are missing for your health care needs?” with available response options that included the following: free/low-cost medical care, mental/behavioral health services, vaccination services, physical or occupational therapy, and others. To assess participants’ *barriers to healthcare access*, we asked, “What are the barriers (if any) that keep you from accessing healthcare when you need it?” Available response options included the following: limited or no health insurance coverage, unable to afford out-of-pocket costs, lack of primary care physicians, lack of reliable transportation, and others. Participants could choose all responses that applied for both questions. Academic and community-based research team members worked together to identify these items as potential needs and barriers for the population of shooting survivors in focus for this study.

Following the survey, the interview guide consisted of a total of 16 open-ended questions that asked interviewees to discuss their childhood experiences and circumstances around their injury. Participants were asked to consider their experiences both pre- and post-injury, their overall health, health habits, and their sense of safety and protection in their current living situation.

### Analytic Strategy

Following best practices for convergent mixed-methods studies, data from the survey and interviews were not analyzed until both data collections were complete. Table A1 in the Appendix highlights the post-data collection integration plan. Data analysis occurred separately and then was merged through an explanatory unidirectional approach, where survey data framed analysis and was enhanced by qualitative experiences across the survey result categories [29, 30]. Given the sample size, the results of the survey are limited to descriptive analysis. We generated descriptive statistics for all demographic measures and then tallied descriptive statistics regarding health challenges (health satisfaction, pain, daily medical treatment needed, and functional disabilities), healthcare needs, and barriers to healthcare access across the full sample. Response numbers resulting from missing answers for all individual items are depicted in the results since survey respondents chose to skip certain questions based on their level of comfort.

We followed a pragmatic approach for qualitative data analysis. The research team, consisting of both university and community researchers, conducted open coding of all 15 transcripts, using line-by-line coding to identify themes. Following this phase, the team met to go through each transcript to discuss themes from the open-coding process. The team created a collaborative codebook based on those themes. A subset of the team then engaged in a focused coding phase to code each transcript by themes aligning with health challenges, healthcare needs, and barriers to access. Our data analysis employed inductive thematic saturation, limited to the level of analysis. Saturation was achieved among the sample following multiple coding rounds, and when no new insights related to healthcare barriers and access arose. The qualitative and quantitative results are presented as integrated findings across key domains of health challenges, healthcare needs, and barriers to healthcare access.

## Results

Table 1 provides demographic information for the 107 survey participants, noting the number of available responses for each measure after accounting for missing answers. The majority of the sample identified as male (81%), Black/African American (67%), and non-Hispanic/Latino (70%). Most respondents (66%) were unemployed with a high school education (57%) or less (30%). The majority of participants reported income of less than $10,000 each year (58%) and most individuals were unmarried and not in a committed relationship (57%). The average age of respondents was 39 years old.

**Table 1:** Sample characteristics

	*N* (%)^a^
Gender identity (*N* = 105)^b^	
Male	85 (81)
Female	16 (16)
Other^c^	11 (11)
Race (*N* = 90)^b^	
Black/African American	60 (67)
White	9 (10)
American Indian/Alaska Native	8 (9)
Asian	2 (2)
Caribbean Black	2 (2)
Native Hawaiian/Pacific Islander	1 (1)
Other	11 (12)
Ethnicity: Hispanic/Latino (*N* = 87)	26 (30)
Unemployed (*N* = 106)	70 (66)
Education (*N* = 106)	
< High school	32 (30)
High school diploma	60 (57)
Associate’s	5 (5)
Bachelor’s	6 (6)
Master’s	3 (3)
Income (*N* = 77)	
< $10 K	45 (58)
$10–$19,999	13 (17)
$20 K–$29,999	7 (7)
$30 K–$69,9999	12 (15)
Relationship status (*N* = 103)	
Unmarried/uncommitted	59 (57)
Unmarried/committed/separate	11 (11)
Unmarried/committed/together	24 (23)
Married^d^	9 (9)
Age (mean) (*N* = 81)	39

^a^Percentages calculated based on available responses

^b^Respondents indicated all categories that apply

^c^Other includes: gender non-binary (0%), transgender man (0%), transgender woman (1%), prefer to self-identify (3%), and prefer not to disclose (7%)

^d^Includes married living together and separate

### Health Challenges

Many of the survivors in our study grapple with physical and mental changes after their injury, often inhibiting activities of daily life or requiring medical treatment. Table 2 depicts the health challenges experienced by survivors. A little more than a quarter of participants said they were either very or fairly dissatisfied with their health, whereas roughly 52% said they were satisfied or very satisfied with their health. About two-thirds of the participants indicated that pain prevents them from doing what they need to do either a moderate amount, a great deal, or an extreme amount. Roughly 22% of the sample said they do not need any amount of medical treatment to function in daily life, while nearly 60% of the sample indicated needing a moderate amount, a great deal, or an extreme amount of medical treatment to function.

**Table 2:** Health challenges among shooting survivors

	*N* (%)^a^	Mean	Min	Max
*Health satisfaction* (*N* = 106)		3.27	1	5
Very dissatisfied	9 (8)			
Fairly dissatisfied	20 (19)			
Neither satisfied nor dissatisfied	21 (20)			
Satisfied	45 (42)			
Very satisfied	11 (10)			
*Pain prevents doing what you need to do* (*N* = 105)		2.76	1	5
Not at all	19 (18)			
A small amount	19 (18)			
A moderate amount	38 (36)			
A great deal	26 (25)			
An extreme amount	3 (3)			
*Amount of medical treatment needed to function* (*N* = 104)		2.71	1	5
Not at all	23 (22)			
A small amount	20 (19)			
A moderate amount	30 (29)			
A great deal	26 (25)			
An extreme amount	5 (5)			
*Functional disabilities* (*N* = 61)^b^				
Hearing	8 (13)			
Vision	16 (26)			
Cognitive	21 (34)			
Ambulatory	13 (21)			
Self-care	9 (15)			
Independent living	15 (25)			

^a^Percentages calculated based on available responses

^b^Respondents indicated all that apply

Participants who responded to questions about functional disabilities (*N* = 61) indicated these disabilities were relatively common. For instance, about 1 in 4 said they experience a visual disability whereas more than a third experience some type of cognitive disability. Approximately 1 in 5 participants indicated trouble walking or climbing stairs (ambulatory disability) while 15% indicated difficulty with self-care (e.g., bathing or dressing). Finally, 1 in 4 participants indicated difficulties with independent living including running errands alone like going shopping or to a doctor’s appointment.

One participant spoke about transitioning from walking to using a wheelchair following their injury, sharing their exercise efforts and daily routine changes. Another participant discussed breathing challenges and activity limitations:

Before I was injured, I was physically fit. Like I was able to, you know, do everything that I wanted to do. Like now, … my doctor’s like, I can’t, he don’t want me to do running because he said, my lung, it’s a chance that it can collapse. So like that [is] slowing me down, going to work ‘cause I can’t, you know - they don’t want me to take the risk of lifting something too heavy or being out there on my own [at work] and something happens and I’m by myself.

Participant narratives discussed changes in physical ability that can also be interpreted as a partial loss of bodily autonomy. Participants discussed how their physical injuries, and this potential loss, were also connected to mental health challenges. After their injury, one participant never used the words “mental health” but rather described their psychological well-being in the form of a dark cloud. Another participant hinted at being depressed but having the ability to not “stay in it for long,” as they were committed to ensuring they could “get out of it” on their own. Two participants recalled specific physical reactions in their bodies post-injury and how their mindsets shifted:

I experienced cold sweats. I think about the shit, and I try not to think about the shit. You feel me? The psychiatrist, she did tell me ‘You may not feel things now or you may not go through things in a couple of years.’ This could be PTSD.

Every time I wake up, I feel like I am going to get shot again. My anxiety is crazy and my mindset is different. Now, I feel like it’s gonna have me going crazy.

Other participants felt that post-injury, their mental health actually became stronger and that their outlook on life was more positive. Two individuals shared:

It didn’t make me depressed, it just made me more on point… I know people who got shot worse. Like, it ain’t like I had to go through therapy or anything like that, or physical therapy. I look at that as a blessing.

My mental health was good before, now it seems a lot stronger. I’m more motivated to do what I have to do ‘cause I know I had a second chance at life. It comes and goes, you know. You go through depression, and I mean I still have PTSD. As long as you stay focused, surround yourself with positive people, positive imaging since we got social media, your mental could be stronger.

### Healthcare Needs

**Fig. 1:**
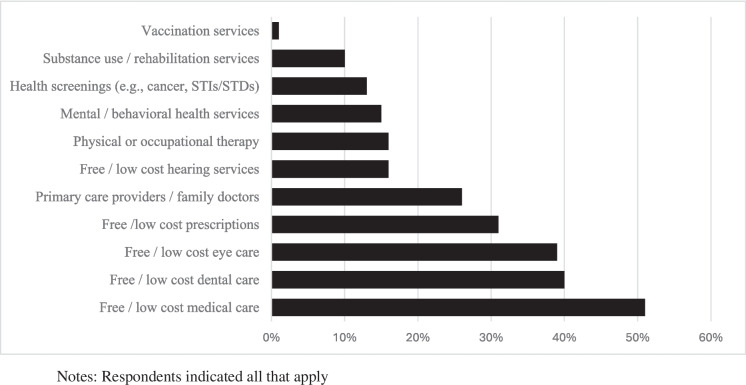
Missing healthcare needs among shooting survivors (*N* = 87)

Figure 1 illustrates the healthcare needs among the 87 participants that responded to the survey prompt. More than half of respondents indicated that they lacked access to free or low-cost medical care, while about 40% indicated a need for specialist services like dental and eye care. Roughly 1 in 4 respondents indicated a need for a primary care provider or family doctor, while about 15% of the sample said they needed access to physical/occupational therapy or mental/behavioral health services.

Qualitative data spoke less directly to the cost burden of healthcare and instead to overall avoidance of the healthcare system. Participants discussed how their injury required them to see a doctor, although many participants pre-injury and at times post-injury did not often seek care. Multiple participants discussed engaging in homeopathic remedies, saying:

I started following my own remedies and guidelines. Like, I used to like watch [medical] videos and stuff. Find like little like herbs and stuff to take. Like, say, if I did have a cold or something, I would drink tea.

…I don’t go to the doctor…I’m straight vegetarian. Like I cure myself. Anything element that I got to do with me, I’m gonna go research it and take care of myself naturally.

These quotes highlight participants’ medical autonomy and may also speak to potential distrust of healthcare institutions or unfamiliarity with how to engage in certain healthcare processes (e.g., follow-up care, finding a specialist).

Others changed their health care habits overall post-injury, one person sharing:

I never used to go to the doctors, never. Like none so far. I ain’t even have a primary doctor or none of that stuff, that type of stuff. But I do now.

Firearm violence has a lasting impact on one’s mental health, and the use of substances and alcohol are common coping mechanisms for some survivors [11]. About 10% of the sample indicated a need for substance use and rehabilitation services, although only a few participants shared that they use alcohol and drug use to cope with their injury recovery. Examples included having a beer after waking up from a nightmare or using marijuana to feel calmer and relaxed.

Multiple participants expressed their infrequent use of pain medication (e.g., aspirin and related over-the-counter medicines prior to injury) pre-injury and many discussed limiting use post-injury, except when injury pain was immense. One participant spoke about avoiding prescribed pain medications because they were afraid of becoming addicted, while another participant shared, “I ain’t really big on pain medicine anyway. Even when they gave me pain medicine, I probably altogether probably took six pills, maybe.” Infrequent pain medication use could also be a rebuke of reliance on external help, instead relying on inner strength to heal from injury.

### Barriers to Healthcare Access

**Fig. 2:**
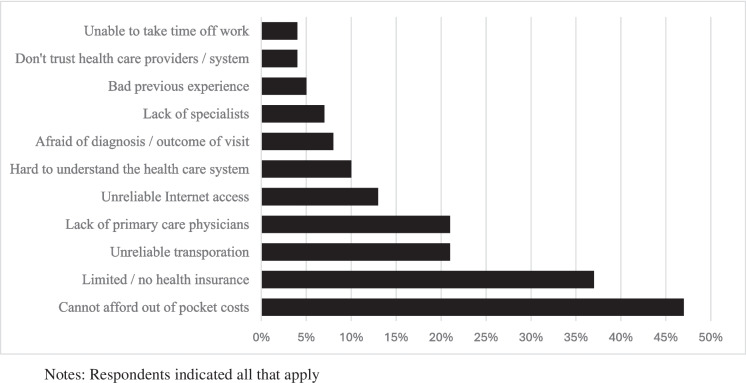
Barriers to healthcare access among shooting survivors (*N* = 92)

Figure 2 shows participants’ barriers to healthcare access among 92 respondents. The most common barrier was the inability to afford out-of-pocket costs (47%), followed by having limited or no health insurance (37%). No qualitative data spoke explicitly about health insurance except for one participant who discussed health insurance as a necessity for their children, but not for themselves. Other significant barriers to healthcare access included unreliable transportation (21%), lack of primary care physicians (22%), and unreliable Internet access (13%).

In the interviews, participants discussed the transportation and logistical help they often needed to attend physical therapy, attend medical follow-ups, and get prescription refills. One person shared:

A lot of stuff could be better inside this healthcare system. Like transportation could be better…when this injury first started and I first started going to the hospital, I missed a lot of appointments due to [transportation agency] not showing up.

Participants also spoke about challenges with refilling prescriptions and finding transportation to pick up medication. One participant mentioned leaving the hospital with pain medication and the challenge of trying to access pain medications once he completed the prescribed dosage, sharing, “They give me medicine for a week so when the week was over, I got to try to call and get back in touch with them [for a refill] and it is just a hassle.” Together, these narratives highlight potential gaps in the continuum of care for patients following injury.

Participants expressed appreciation for the community violence prevention specialists who offered social support and completed logistical tasks with them, such as driving them to appointments, running errands, and helping them fill out paperwork post-injury. While assisting a participant at the hospital, a CVPS offered their advice, sharing, “’Cause you can call me anytime, bro. Seriously.” The participant responded, “No, I know. And I’ve called you before, and you don’t be quick to hang up or nothing like most people do. You be talking. I realize that from the hospital.”

About 10% of participants said they could not access care because it was hard to understand the healthcare system, whereas about 8% said they are afraid of the outcome of a healthcare visit. Shooting survivors we spoke with engaged with the health care system often involuntarily through transport to a trauma center and through various follow-up appointments and surgeries.

## Discussion

This study was developed through a partnership between university researchers and community-based violence prevention specialists to study the health-related needs of firearm injury survivors in a small Northeastern city. The findings from our survey of 107 shooting survivors provided insight into their own health challenges, access to healthcare, changing health behaviors, and barriers to care. Our qualitative findings highlight specific health challenges faced by survivors, often converging with the broader findings from the survey. In general, we found that shooting survivors face significant health-related challenges, although we uncovered important heterogeneity suggesting that survivor experiences with health and well-being are not monolithic.

Although we are careful to note that our sample is not representative of all shooting survivors due to the purposive sampling approach used in this community-based study, we underscore the significant socioeconomic disadvantage that characterizes the majority of the sample. Most of the survivors were unemployed, had a high school diploma or less, and annual income of less than $10,000. The majority of respondents were unmarried and uncommitted, while a significant portion said they do not have access to free or low-cost medical care or have limited/no health insurance. In general, shooting survivors in this sample were found to live in financially precarious circumstances with rather tenuous access to consistent yet needed medical care.

Those who have sustained firearm injuries contend with health concerns related to pain, mental health, and everyday functional ability. For instance, the quantitative findings reflect that a majority of shooting survivors have a moderate or great level of pain that impacts their ability to function. Relatedly, many of the participants stated needing at least a decent amount of medical treatment to function. Additionally, it was not uncommon for participants to have a significant disability such as cognitive impairments, vision impairments, or an inability to function independently. Some participants may have experienced a sense of loss of their own bodily autonomy, and a new reliance on other people and medications to assist them. In interviews, participants discussed mental health concerns such as depression or PTSD. On the other hand, some of the survivors discussed feeling like they had been given a second chance at life or felt fortunate compared to others who were in a worse situation. Like those who have been incarcerated and discover a new lease on life through self-defined redemption narratives [31, 32], these interview responses suggest that some survivors re-interpret their own ordeals in a positive light, helping to improve their well-being and outlook for the future.

When trying to access health care, participants noted barriers such as being able to afford out-of-pocket expenses, needing health insurance, transportation (both reliability and frequency of the need), the lack of primary care physicians, and internet access. Participants responded that resources/services that were missing included free/affordable medical care, dental care, and prescriptions. The results also reflect that many participants have decided to take care of their health without assistance from the broader healthcare establishment, including the use of homeopathic remedies for some and coping through substance use for others. Relatedly, some participants desired medical independence and expressed that they could cure themselves, echoing prior research showing that shooting survivors might see limited benefit to healthcare services, distrust practitioners, and fear the stigma that may accompany care seeking [33]. As a whole, though, few participants seemed to report outright distrust of the healthcare system on the survey and some participants even discussed positive interactions with the healthcare system. However, accessing prescriptions and fear of addiction were concerns.

These findings have implications for healthcare delivery among shooting survivors. Given the often extensive health consequences of firearm injuries for survivors, treatment plans should include a discussion about short-term and long-term needs. These plans should also take into consideration logistical issues that may arise for shooting survivors, including limited or loss of physical independence, medication needs, financial barriers, and transportation to appointments and pharmacies. Additionally, healthcare providers should be aware that concerns about addiction may impact one’s decision to adhere to medication regimens [34]. Non-healthcare providers including social service workers and community violence prevention specialists can play an important role in assisting with healthcare treatment and medication adherence by assisting with logistical needs (e.g., transportation to pharmacies, office appointments, or locations with Internet to complete paperwork).

Assistance with the mundane but vital barriers to care for shooting survivors can be supported by hospital-adjacent violence prevention specialists working in hospital-based violence intervention programs (HVIP’s). However, it is critical to ensure that HVIP intervention services on offer align with the needs of clients, including some of those illustrated in the current study [35]. Enhanced co-location of medical and social services through HVIP programs and other models such as Trauma Recovery Centers for shooting survivors can support better treatment adherence while reducing barriers to care to improve long-term outcomes [36]. Our findings reinforce the notion that systemic inequities related to socioeconomic disadvantage and lack of access to healthcare intersect with patterns of firearm violence to exacerbate health inequities [37]. Service provision and post-injury care for shooting survivors must therefore explicitly consider the broader set of social determinants of health to develop the best recovery plan for individuals towards the aim of reducing inequitable outcomes among high-need populations.

There are several limitations to the current study that offer opportunities for future research. First, although we were able to conduct surveys with 107 shooting survivors, a significant challenge given the difficulty of reaching this population, this is a descriptive study. The results are not representative of all firearm injury survivors, but rather reflective of purposive sampling in a single city driven by the research team’s emphasis on community-based recruitment. In particular, our sampling procedure likely overrepresents survivors connected to service provider networks, limiting the generalizability of the findings especially regarding barriers to care among those not actively engaged with community partners and provider resources. We encourage researchers to continue assessing the health needs and barriers to care among firearm injury survivors, as well as opportunities for enhanced intervention, using broader and more representative data to the extent possible.

Second, respondents were included in our study regardless of when they had been injured with a firearm. Since many respondents did not want to discuss the details of their injury, we could not know how recently someone had been shot. In some cases, it was clear that injuries were quite recent, while in others respondents disclosed that the shooting happened years before the study was conducted. This should be assessed in future studies to the extent feasible.

Third, our study focused predominantly on physical health given that past work has often prioritized the study of mental well-being. Although we investigated practical barriers to healthcare access alongside physical health challenges, future studies would benefit from assessing other outcomes including perceptions of safety, impacts on education and employment, and interactions with the healthcare system at the time of injury.

Despite these limitations, our mixed methods study offers insight into a variety of health challenges, healthcare needs, and barriers to care among firearm injury survivors. The results were not uniform across outcomes, a reminder that the needs of firearm injury survivors are often complex and multifaceted. Our community-informed, participatory approach emphasized collaboration between university researchers and community violence prevention specialists, yielding findings that illustrate the need for comprehensive care both within and beyond healthcare systems. There remains a vital need to systematically assess the healthcare and support needs of firearm injury survivors across the U.S. to inform effective treatment approaches and guide prevention efforts aimed at mitigating the national burden of gun violence in the USA.

## Supplementary Information

Below is the link to the electronic supplementary material.

ESM 1 (DOCX 112 KB)

## Data Availability

Data is available upon reasonable request from the corresponding author, Dr. Daniel Semenza.
