# Supplementary material for: Physical Health Challenges, Healthcare Needs, and Barriers to Care among Firearm Injury Survivors: A Mixed Methods Analysis
Source: J Urban Health. 2026 Jan 26;103(1):55–65. doi: 10.1007/s11524-025-01049-9 (PMC13136478; doi:10.1007/s11524-025-01049-9)
Supplement: Supplementary file 1 — (DOCX 112 KB) [file 11524_2025_1049_MOESM1_ESM.docx]

**Appendix**

**
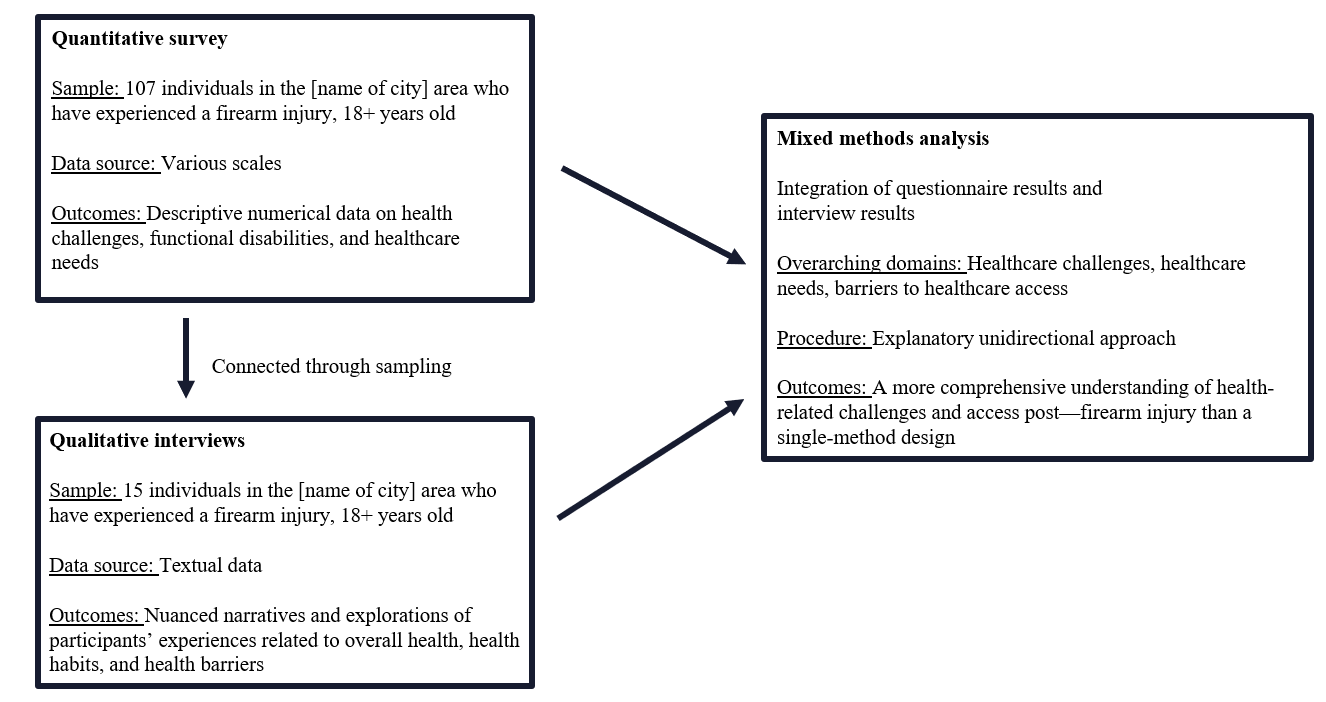
Figure A1.** Mixed Methods Convergent Design

**Table A1.** Joint Display of Data Collection for Integration

| **Domain** | **Quantitative data source & items**  **(**Survey with 107 respondents) | **Qualitative Data source & item**  **(**Interviews with 15 participants ) |
| --- | --- | --- |
| **Healthcare Challenges** | Select questions from World Health Organization’s (WHO) Quality of Life – Brief Scale (WHOQOL-BREF)   - How satisfied are you with your health? - To what extent do you feel that physical pain prevents you from doing what you need to do? - How much do you need medical treatment to function in your daily life? | **Mental health**   - How would you describe your mental health throughout your life/ overall? - In what ways have you experienced any changes in your mental health after your injury? - What do you do to cope with these feelings?   - Did you ever find yourself using substances to cope? - Have you experienced any flashbacks, nightmares or changes in mood? - Have you or would you seek mental health services from a therapist, psychologist or psychiatrist? Why or why not?   **Physical health**   - How would you describe your physical health throughout your life/overall? In what ways has your physical health changed after your injury?   - Did your injury cause any new health problems, or make any prior health conditions worse? additional health problems? If so, what are they? - In what ways have your new health issues impacted your life post injury? |
|  | Six items derived from the American Communities Survey and the US Census Bureau related to functional disabilities  Are you currently experiencing any of the following functional difficulties?  (1) hearing difficulty: deafness or having serious difficulty hearing  (2) vision difficulty: blindness or having serious difficult seeing, even when wearing glasses  (3) cognitive difficulty: having difficulty remembering, concentrating, or making decisions  (4), ambulatory difficulty: having serious difficulty walking or climbing stairs  (5) self-care difficulty: having difficulty bathing or dressing, and  (6) independent living difficulty: having difficulty doing errands alone such as visiting a doctor’s office or shopping. |  |
| **Healthcare Needs** | What are the resources or services you think are missing for your health care needs? (select all responses of 12 options e.g., health screenings, , physical or occupational therapy, primary care providers, free/low cost prescriptions etc.) | **Experiences with the healthcare system**   - How would you describe your experiences or relationship with the healthcare system throughout your life/overall? - What happened when you got to the hospital? Tell me about your hospital stay. - What was your experience like in the hospital after your injury?   - How did the staff treat you? - How could the hospital services be changed to meet your needs? |
| **Barriers to Healthcare Access** | What are the barriers that keep you from accessing healthcare when you need it?  (select all responses of 13 options e.g., don’t trust health care providers, unreliable transportation, cannot afford out-of-pocket costs) | **Access to health care resources**   - Can you talk a little bit about the doctors and/or type of health care you are using after your injury (e.g., physical therapist, occupational therapist)   - How has your experience been with these types of providers/doctors following the injury?   **Disability**   - Did your injury cause you to become disabled? If so, how? - What resources were you able to access because of your disability? |
